# Targeted Nanoparticles for Pediatric Leukemia Therapy

**DOI:** 10.3389/fonc.2014.00101

**Published:** 2014-05-13

**Authors:** Riyaz Basha, Nirupama Sabnis, Kenneth Heym, W. Paul Bowman, Andras G. Lacko

**Affiliations:** ^1^Department of Pediatrics, University of North Texas Health Science Center, Fort Worth, TX, USA; ^2^Institute for Cancer Research, University of North Texas Health Science Center, Fort Worth, TX, USA; ^3^Departments of Integrated Physiology and Pediatrics, University of North Texas Health Science Center, Fort Worth, TX, USA; ^4^Cook Children’s Medical Center, Fort Worth, TX, USA

**Keywords:** leukemia, nanoparticles, drug delivery systems, targeting, high density lipoprotein

## Abstract

The two major forms of leukemia, acute lymphoblastic leukemia (ALL) and acute myeloid leukemia (AML), account for about one-third of the malignancies diagnosed in children. Despite the marked successes in ALL and AML treatment, concerns remain regarding the occurrence of resistant disease in subsets of patients, the residual effects of therapy that often persist for decades beyond the cessation of treatment. Therefore, new approaches are needed to reduce or to avoid off target toxicities, associated with chemotherapy and their long-term residual effects. Recently, nanotechnology has been employed to enhance cancer therapy, via improving the bioavailability and therapeutic efficacy of anti-cancer agents. While in the last several years, numerous review articles appeared detailing the size, composition, assembly, and performance evaluation of different types of drug carrying nanoparticles, the description and evaluation of lipoprotein-based drug carriers have been conspicuously absent from most of these major reviews. The current review focuses on such information regarding nanoparticles with an emphasis on high density lipoprotein-based drug delivery systems to examine their potential role(s) in the enhanced treatment of children with leukemia.

## Molecular Pathogenesis of Leukemias

Cancer is a major contributor to disease related deaths among 1–19-year old children (1, 2). The incidence of childhood cancer has been slowly increasing during the past four decades as more than 12,000 children are diagnosed annually with various types of malignancies in the US (3, 4). Leukemias are classified based on the type of white blood cell (myeloid or lymphoid) and the degree of maturity and proliferative tendencies (acute or chronic) of the cell populations involved. The most common leukemias in children are Acute Lymphoblastic Leukemia (ALL) and Acute Myeloid Leukemia (AML); while ALL is responsible for the majority of pediatric cancer-related deaths (5–8).

Current treatment strategies for leukemia involve chemotherapy, bone marrow transplant, and radiation. These treatments often induce long-term side-effects, resulting in impairment of vital physiological functions among the survivors (9). While current treatment approaches have greatly improved the prognosis for survival for pediatric leukemia, some patients remain refractive to current therapeutic regimens (10, 11). Hence there is an urgent need for novel therapeutic strategies for these difficult to treat leukemia cases, in addition to reducing the long-term impact of therapy (residual side-effects) for all leukemia patients.

## Targeted Therapies in Treating Leukemia

Due to the cytotoxicity of drugs, currently the major challenge is to deliver the therapeutic agent to neoplastic cells while preserving the viability of non-malignant cells. In some cases, the efficacy of therapeutic agents are greatly reduced due to acquired resistance by cancer cells. Targeted therapies are currently under development to address resistance to therapy and to reduce unwanted side-effects. Research on the use of nanoparticles as drug carriers has advanced to the point to focus on assessing the safety and efficacy of such drug delivery systems (12).

## Nanoparticles in Targeted Therapy

The application of nanotechnology is rapidly advancing toward treating several major diseases including cancer. Selective delivery of anti-cancer agents to cancer cells without harming the healthy cells is a major goal of these current efforts. In order to achieve these goals, small (nano) particles as drug carriers have been employed, specifically to target (malignant) malignant cells and tumors (13–16). The utilization of nanoparticles as drug delivery agents has several advantages, including specific targeting via receptor mediated mechanisms and effective penetration of the tumor microenvironment. The use of nanotechnology in cancer treatment has gained significant momentum since 1995 in the US when the FDA approved the use of a nano-drug, Doxil, a liposomal delivery formulation of a cardioprotective form of Doxorubicin. Doxil has been developed for increasing the bioavailability and extending the drug’s residence time in the circulation, with subsequent release at the tumor site (17). Subsequently, despite meticulous screening and extremely low approval rates by the FDA (18), a few nanoparticles/carriers including Abraxane and Marqibo have been approved for cancer treatment. Abraxane, an albumin-bound paclitaxel formulation has been approved for some metastatic and relapsed breast cancer cases in 2005. Later, this drug was also approved for treating locally advanced or metastatic non-small cell lung cancer in 2012 and for treating late-stage pancreatic cancer in 2013. Marqibo, a liposomal formulation of vincristine has been granted FDA expedited approval in 2012 for certain Philadelphia chromosome-negative (Ph)-ALL patients.

## Pediatric Leukemias and Nanotherapeutics

Despite recent improvements in the prognosis for patients with ALL and AML, serious concerns remain regarding off target toxicity and residual effects of chemotherapy (19–21). Several strategies including the use of nanoparticles have been proposed to avoid these concerns including its long-term impairment of physiological functions (22). A variety of nanoparticles, including dendrimers, gold particles, liposomes, micelles, and polymers have been described to improve the bioavailability or the therapeutic efficacy of currently used anti-cancer agents (14, 16). These nanodelivery systems have yet to be evaluated in pediatric leukemia patients.

## Lipoprotein-Based Drug Carriers in Cancer Therapy

Advances in the last 10 years have brought the outstanding features of lipoprotein drug delivery systems to the forefront of experimental therapeutics (23). Lipoproteins possess key characteristics that allow them to perform as superior drug delivery agents. Lipoproteins contain a sealed core compartment that protects the encapsulated pharmaceutical agent from being rapidly taken up by normal tissues (or to elicit an antigenic response) upon entering the blood circulation. Once entering the blood, the delivery of the drug from the lipoprotein nanoparticle may be facilitated by receptor/ligand interactions via the polypeptide/apolipoprotein component of the nanocomplex (24, 25).

## Leukemia/Lymphoma Therapeutics via Lipoprotein-Based Drug Delivery Systems

Vitols et al. found that leukemic cells with monocyte differentiation expressed an elevated low density lipoprotein (LDL) receptor (26). Subsequently, Vitols et al. established that human leukemia cells were avidly taking up LDL and thus proposed lipoproteins as potential drug transporting vehicles for selective leukemia therapeutics (27). More recently, Masquelier et al. attempted to use LDL (28) as a vehicle for delivering chemotherapeutic agents to leukemia cells. While these studies have shown some promise in improving the bioavailability of lipophilic drugs the stability of the drug carrying LDL complexes did not meet expectations (29). While the LDL carrier has not so far shown sufficient promise for advancement toward clinical applications, the studies of Vitols et al. have drawn attention to lipoproteins and their receptors as components of a selective drug delivery strategy for cancer chemotherapy (26, 27). Recently, Yang et al. showed that human lymphoma cells overexpressed the high density lipoprotein (HDL)/SR-B1 receptor while normal lymphocytes had low baseline expression (24). Our studies have shown that the great majority of malignant cells and tumors also overexpress this receptor (30) and thus validate the use of HDL type nanoparticles in the cancer cell/tumor selective delivery of anti-cancer agents (31).

**Figure 1:**
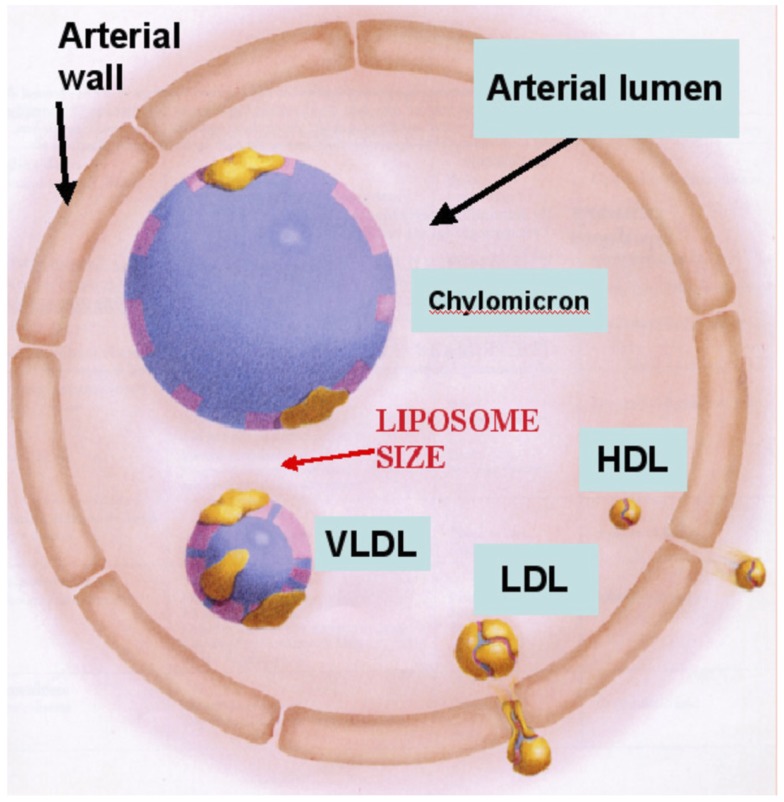
**Size comparison of HDL with liposomes and other lipoprotein classes**. The rHDL nanoparticles have an average diameter of 14 nm, similar to native (circulating HDL) and thus are likely to gain easier access to cancer cells than other drug carriers, including liposomes.

The features of rHDL nanoparticles that qualifies them as promising drug delivery vehicles include: their small size (Figure 1), stability, and receptor mediated cellular uptake of their core components (23, 25, 30, 31). Additional major advantages of rHDL as a drug delivery vector include: (1) natural components, providing enhanced safety and efficacy; (2) stable non-leaking preparation; (3) biocompatibility, several formulations with the same ingredients (as rHDL) have been safely injected into human subjects (32–35); (4) HDL particles exhibit longer residence time in the circulation than most drug formulations or other lipoproteins because of their property to escape reticulo-endothelial system; (5) the small size of the rHDL nanoparticle (Figure 1) could be a major advantage in leukemia therapeutics as the surface exposure of the nanoparticles and their interactions with leukemia cells would be markedly enhanced. Consequently, it is anticipated that the delivery of anti-leukemia agents to leukemic cells would be substantially enhanced.

## Selective Delivery Feature of rHDL Nanoparticles to Malignant Cells

Because of the substantial overexpression of the HDL/SR-B1 receptor by most cancer cells (23–25, 30, 31) and tumors (30), the rHDL nanoparticles are capable of selectively delivering their therapeutic payloads to malignant cells and tumors without impacting most normal tissues (23, 30, 31). This feature of the rHDL nanoparticles is a major advantage over non-targeted delivery systems as it is anticipated to substantially reduce off target toxicity to normal tissues, a major concern during the treatment of pediatric cancer patients.

## Rationale for the Use of rHDL Nanoparticles in Leukemia Therapeutics

In addition to the above description of the rHDL nanoparticles, there are more compelling reasons for their utility as drug carriers during leukemia chemotherapy. Clinical studies have supported the concept of targeting cancer cells and tumors via lipoprotein carriers. A case–control study of 519 patients with various types of solid tumors and 928 controls reported that the serum lipid total cholesterol values of patients were significantly lower, due primarily to low levels of HDL-C (36). The findings of these and other studies are consistent with the concept that lipoprotein receptors (especially the HDL receptor) are highly active on the surface of malignant cells (25) and thus may be used as conduits for the delivery of anti-cancer agents, including those for leukemia therapy. Clinical studies have consistently shown a reduction of HDL-C levels in ALL patients (37). The HDL-C values returned to normal levels once the patients were in remission (38). The rHDL nanoparticles deliver anti-cancer agents to cancer cells and tumors via a receptor mediated mechanism that provides a robust selective targeting vehicle during chemotherapy. The receptor mediated uptake of anti-cancer agents is an important part of the concept for efficient drug delivery because leukemia cells are likely to have a high expression of the SR-B1 receptor compared to normal cells, due to the excessive need for cholesterol to support their high rate of proliferation. This may be particularly potent in cells that have a very high mitotic rate.

## Conclusion

Nanotechnology is providing tools for effective use of materials at a very small scale. This emerging technology utilizes multidisciplinary approaches suitable for biomedical applications in health care including diagnosis and therapy. We anticipate that receptor mediated uptake of anti-cancer agents (25, 31) can function as an efficient drug delivery system for leukemia therapy. Leukemia cells are likely to exhibit high expression of the SR-B1 receptor. Hence we postulate that our model could be very effective and even serve as a personalized treatment strategy. By determining the pre-treatment expression level of these receptors in individual patients, the chemotherapeutic regimen could be personalized for maximum benefit specifically to the high SR-B1 expressing leukemia cells and tumors. Alternatively for leukemia cells and tumors exhibiting low SR-B1 expression, functionalized rHDL nanoparticles could be developed to target a wide range of *other surface tumor antigens*. There are a multitude of membrane components available as possible targets for functionalized rHDL, which can be easily modified to feature the desired vector for targeting (39). This strategy would reroute the lipoproteins from their natural receptors and navigate them to malignant cells and tumors via other receptors.

The targeted therapy via the rHDL nanoparticles is anticipated to limit the toxicity of drugs during therapy, via a novel drug delivery model with desirable physical characteristics and outstanding selective targeting potential toward malignant cells and tissues (20–22, 25, 28). Additionally, the rHDL delivery system has a potential to reposition a variety of approved drugs that proved to have limited applicability due to poor solubility and excessive peripheral toxicity. This approach could be most economical and profitable, in contrast with conventional and very costly drug and target-screening strategies.

## Conflict of Interest Statement

The authors declare that the research was conducted in the absence of any commercial or financial relationships that could be construed as a potential conflict of interest.
